# 异基因造血干细胞移植治疗伴TP53基因异常骨髓增生异常综合征/急性髓系白血病42例疗效分析

**DOI:** 10.3760/cma.j.issn.0253-2727.2023.03.008

**Published:** 2023-03

**Authors:** 丹 冯, 铭洋 王, 佳 刘, 海啸 张, 欣 陈, 荣莉 张, 卫华 翟, 巧玲 马, 爱明 庞, 栋林 杨, 嘉璘 魏, 祎 何, 四洲 冯, 明哲 韩, 尔烈 姜

**Affiliations:** 中国医学科学院血液病医院（中国医学科学院血液学研究所），实验血液学国家重点实验室，国家血液系统疾病临床医学研究中心，细胞生态海河实验室，天津 300020 State Key Laboratory of Experimental Hematology, National Clinical Research Center for Blood Diseases, Haihe Laboratory of Cell Ecosystem, Institute of Hematology & Blood Diseases Hospital, Chinese Academy of Medical Sciences & Peking Union Medical College, Tianjin 300020, China

**Keywords:** 骨髓增生异常综合征, 急性髓系白血病, 基因，TP53, 异基因造血干细胞移植, Myelodysplastic syndromes, Acute myeloid leukemia, TP53 gene change, Allogeneic hematopoietic stem cell transplantation

## Abstract

**目的:**

探讨异基因造血干细胞移植（allo-HSCT）治疗伴TP53基因异常骨髓增生异常综合征（MDS）/急性髓系白血病（AML）患者的疗效及影响因素。

**方法:**

纳入2014年8月1日至2021年7月31日期间于中国医学科学院血液病医院接受allo-HSCT的42例伴TP53基因异常的MDS/AML患者，根据TP53异常情况分为TP53缺失组、TP53单基因突变组和TP53多打击组，回顾性分析各组患者的临床特征、移植疗效及其预后影响因素。

**结果:**

全部42例患者中包括MDS、AML患者各21例，中位移植年龄为41.5（18～63）岁，中位随访时间为34.0（7.5～75.0）个月。移植后3年总生存（OS）率为66.3％（95％*CI* 53.4％～82.4％），3年无事件生存（EFS）率为61.0％（95％*CI* 47.7％～78.0％）。TP53缺失组、TP53单基因突变组、TP53多打击组移植后3年OS率分别为82.5％（95％*CI* 63.1％～100.0％）、60.6％（95％*CI* 43.5％～84.4％）、57.1％（95％*CI* 30.1％～100.0％）（*χ*^2^＝3.100，*P*＝0.200），EFS率分别为80.8％（95％*CI* 60.0％～100.0％）、55.0％（95％*CI* 37.8％～80.3％）、50.0％（95％*CI* 25.0％～100.0％）（*χ*^2^＝0.900，*P*＝0.600）。单因素分析发现，年龄、复杂核型、共突变、移植前原始细胞阳性、移植后原始细胞阳性为OS、EFS的共同预后不良因素。多因素分析显示移植前原始细胞阳性是OS的独立危险因素（*P*＝0.037，*HR*＝33.40，95％*CI* 1.24～901.17）。

**结论:**

allo-HSCT可改善伴TP53基因异常MDS/AML患者的不良预后，但伴有复杂核型的TP53异常患者预后较差。移植前末次流式细胞术检测骨髓原始细胞对预后评估具有一定的指导意义。

## Abstract (en)

**Objective:**

TP53-abnormal MDS/acute myeloid leukemia（AML）patients' allogeneic hematopoietic stem cell transplantation（allo-HSCT）treatment's effectiveness and influencing factors should be studied.

**Methods:**

42 patients with TP53 gene status change MDS/AML who underwent allo-HSCT from 2014.8.1 to 2021.7.31 at the Hematology Hospital of the Chinese Academy of Medical Sciences were the subject of a retrospective analysis. The 42 patients were divided into three groups: the TP53 deletion group（group A）, TP53 mono-alle mutation group（group B）, and TP53 multi-hit group（group C）. The differences in clinical features and prognostic factors after transplantation were analyzed.

**Results:**

There were 42 MDS/AML patients, including 21 patients with MDS, and 21 patients with AML. The median follow-up period was 34.0（7.5–75.0）months and the median patient age at the time of transplantation was 41.5（18–63）years old. The total OS was 66.3％（95％ *CI* 53.4％–82.4％）in 3 years after transplantation, and EFS was 61.0％（95％ *CI* 47.7％–78.0％）in 3 years. For 3 years after receiving hematopoietic stem cell transplantation, there were no statistically significant differences in 3-year OS and EFS in groups A, B, and C（*P*≥0.05）. The 3 years OS was 82.5％（95％ *CI* 63.1％–100.0％）in group A, 60.6％（95％ *CI* 43.5％–84.4％）in group B, and 57.1％（95％ *CI* 30.1％–100.0％）in group C. Univariate analysis revealed that the number of co-mutant genes, pre-HSCT treatment, and disease type did not affect prognosis, while age, karyotype, co-mutation, positive blast cell before transplantation, and positive blast cell after transplantation were common prognostic factors for OS and EFS（*P*<0.1）. MRD levels before transplantation were found to be independent risk factors for OS（*P*＝0.037, *HR*＝33.40, 95％ *CI* 1.24–901.17）in a multivariate analysis.

**Conclusion:**

Patients with MDS/AML who have TP53 mutations can benefit from allo-HSCT, but patients with complex karyotypes have a worse prognosis. Meanwhile, the final flow cytometry（FCM）monitoring blast cell test before HSCT has a certain guiding significance for prognostic assessment.

TP53基因突变是恶性肿瘤性疾病中常见的基因突变之一[1]–[3]。TP53基因是一种抑癌基因，定位于17号染色体短臂1区3带第1亚带（17p13.1），编码P53转录蛋白这一重要的肿瘤抑制因子[4]。TP53基因在正常细胞中低表达，具有诱导细胞周期停滞、促进DNA修复、诱导凋亡、抗衰老、自噬、抗氧化等作用[5]，通过阻滞致癌基因突变的累积来调控肿瘤细胞的增殖，发挥抑癌基因功能。TP53基因状态改变及相应染色体缺失是多种肿瘤的不良预后因素[6]。相关研究显示P53蛋白的表达水平可预测多发性骨髓瘤患者的生存[7]。TP53基因突变在骨髓增生异常综合征（MDS）中的发生率为8％～12％，在急性髓系白血病（AML）中的发生率约为8％[8]，随着分子遗传学、细胞遗传学机制的深入研究，TP53基因突变、17号染色体/17号染色体长臂缺失（−17/17p−）均被美国国立综合癌症网络（NCCN）指南归入AML高危预后因素。NCCN指南将TP53基因突变列为MDS常发生的体细胞突变，作为MDS发生的证据及不良预后因素。有研究发现TP53单等位基因改变患者与野生型患者在临床预后和治疗有效率上没有差异，而TP53多打击改变的MDS患者具有更高的AML转化率以及较差的临床预后[9]–[10]。在AML相关研究中发现，TP53基因突变似乎更易发生在治疗相关AML（t-AML）和伴有骨髓发育不良相关变化AML中，与复杂的细胞遗传学异常、高龄、化疗耐药和不良预后有关[11]。靶向TP53及其通路的药物研发及新治疗方案的应用似乎给伴TP53突变MDS/AML的治疗带来了希望，但目前allo-HSCT仍是临床治愈此部分MDS/AML患者的唯一手段，但allo-HSCT治疗伴不同TP53基因异常患者的疗效尚需进一步探讨。本研究旨在探讨异基因造血干细胞移植（allo-HSCT）对于伴有TP53基因异常（缺失、单等位基因突变、多打击）MDS/AML患者的疗效及其预后影响因素。

## 病例与方法

一、病例来源

本研究对2014年8月1日至2021年7月31日期间于中国医学科学院血液病医院接受allo-HSCT的伴TP53基因异常MDS及AML患者进行回顾性分析。纳入标准：①MDS患者符合2016年WHO诊断分型标准，AML患者符合2017年WHO诊断分型标准；②初诊或疾病进展时染色体荧光原位杂交（FISH）、基因突变分析和（或）传统G显带（CBA）检测到17号染色体改变和（或）TP53基因突变和（或）TP53基因缺失；③接受allo-HSCT并完成规律随访。根据患者TP53基因状态分为三组：①TP53缺失组：仅表现为FISH检测阳性（即仅伴有TP53缺失）；②TP53单基因突变组：仅伴有1个TP53等位基因位点突变；③TP53多打击组：包括伴有−17/17P、不伴有−17/17P但伴有>1个TP53基因位点突变、基因位点突变和TP53缺失并存。

二、检测方法

1. 基因突变检测：①二代基因测序（NGS）检测，提取送检标本（骨髓）中基因组DNA，分析基因蛋白质编码区域的突变，平均测序深度为1 000×，灵敏度范围为1％～5％。结果首先采用Ion Reporter软件进行注释，随后使用多个权威数据库进行筛选，对突变进行注释分析。具体方法参照文献[12]。②FLI3-ITD、NPM1-Exon12、DNMT3A、c-KIT等基因采用PCR方法检测。③CEBPA-TAD、CEBPABZIP、CALR-EXON9、MPL-Exon10、ABL激酶等同时采用sanger测序。

2. TP53基因缺失检测：通过染色体荧光原位杂交（FISH）-P53/CEP17检验，采用CYTOCELL双色标记TP53探针，TP53（17p13）基因标记为红色（R），CEP17（17p11-q11）基因标记为绿色（G），正常信号特征为2R2G，阳性信号特征为2G1R，−17为1R1G，阈值<2.89％为阴性，否则提示缺失。

3. 染色体核型检测：取患者骨髓标本进行常规细胞遗传学分析，采用G显带分析（GBA）技术，每个患者至少分析20个中期分裂相。

4. 骨髓原始细胞流式细胞术（FMC）检测：采用多参数流式细胞术对患者治疗后不同时间点的骨髓标本进行检测，检测敏感度为10^−4^～10^−5^。流式细胞术未检测到肿瘤细胞定义为原始细胞阴性，阳性病例判断标准参照阴性对照及内对照。

三、造血干细胞移植预处理方案

预处理方案均采用清髓性预处理（MAC）方案。21例MDS患者采用以白消安（Bu）+环磷酰胺（Cy）+地西他滨（DAC）+氟达拉滨（Flu）+阿糖胞苷（Ara-C）/去甲氧柔红霉素（IDA）为基础的改良预处理方案；19例AML患者采用以Bu+Cy±IDA/Ara-C为基础的预处理方案，其中2例患者方案中包含克拉屈滨，2例包含美法仑（MEL）；2例AML患者采用TBI+CY方案。单倍型移植、无关供者移植在此基础上加用抗胸腺细胞球蛋白（ATG）。

四、移植物抗宿主病（GVHD）的预防及治疗

采用环孢素A（CsA）或他克莫司（FK506）联合短疗程甲氨蝶呤（MTX）方案预防GVHD，加用或不加用霉酚酸酯（MMF）。GVHD的治疗一线首选方案为糖皮质激素，二线方案包括钙调磷酸酶抑制剂（他克莫司、西罗莫司、CsA等）、芦可替尼、间充质干细胞输注、抗CD25单抗（巴利昔单抗）等。

五、其余移植相关并发症的预防

移植前采用复方丹参滴丸、肝素（PLT>5×10^9^/L）预防肝静脉闭塞病（VOD），膦甲酸钠或缬更昔洛韦等预防巨细胞病毒（CMV）感染，使用复方磺胺甲恶唑预防卡氏肺孢子菌感染，阿苯达唑清除肠道寄生虫，利福昔明预防肠道感染。

六、相关诊断标准和定义

MDS、AML的诊断标准分别参照2016和2017年WHO分型诊断标准。粒细胞植入：中性粒细胞绝对计数（ANC）>0.5×10^9^/L连续3 d；血小板植入：PLT>20×10^9^/L持续1周且脱离血小板输注。GVHD的诊断参照NIH共识及中国专家共识。移植前原始细胞阳性定义为allo-HSCT前最后一次FMC检测结果阳性，移植后原始细胞阳性定义为造血干细胞回输至随访终点前期间内任意一次FMC检测阳性；WT1移植前阳性定义同移植前原始细胞阳性。复杂核型（CK）：在AML/MDS患者的染色体中期分裂相中出现≥3种无相关染色体异常，同时未检出特异染色体异常。

七、随访、研究终点及定义

随访资料来自门诊/住院病历及电话随访。随访截止日期为2022年3月31日。研究终点包括总生存（OS）、无事件生存（EFS）。OS：造血干细胞回输结束至末次随访或死亡的时间。EFS：造血干细胞回输至复发、死亡或末次随访的时间。42例患者中位随访时间为34.0（7.5～75.0）个月。

八、统计学处理

采用R-4.2.0统计学软件进行统计学分析。计数资料采用“频数”和“百分比（％）”表示，正态分布计量资料采用“均值±标准差”表示，非正态分布计量资料采用“中位数（最小值～最大值）”表示。计量资料采用Mann-Whitney检验或ANOVA检验；计数资料采用Chi-square检验或Fisher's exact检验。OS和EFS的分析采用Kaplan-Meier方法，组间比较采用Log-rank检验。将单因素分析中*P*<0.1的因素纳入多因素分析，OS和EFS的多因素分析采用Cox回归，以双侧*P*<0.05为差异有统计学意义。

## 结果

一、伴TP53异常MDS/AML患者的临床特征

共纳入42例伴TP53异常的MDS/AML患者，男30例，女12例，中位移植年龄为41.5（18～63）岁，其中TP53缺失组11例（26.2％）、TP53单基因突变组23例（54.8％）、TP53多打击组8例（19.0％）。42例患者中MDS、AML患者各有21例，三组疾病构成比差异无统计学意义（*P*≥0.05），初诊时血细胞计数及确诊时骨髓原始细胞计数差异均无统计学意义（*P*≥0.05）。42例患者中9例（25.0％）为复杂核型，3组差异无统计学意义（*P*≥0.05）。42例患者中31例（73.8％）检出TP53基因突变，主要位于DAN结合区（DBD），主要突变类型为错义突变（84.21％）。除TP53基因突变外，在患者群体中还发现54种其他基因突变，人均检出3.67个基因突变，突变检出率较高的是ASXL1（8例，19.05％）、RUNX1（7例，16.67％）、TET2（6例，14.29％）、DNMT3A（6例，14.29％）。所有患者中同时伴有1～2个基因突变的患者11例（26.2％），三组间差异无统计学意义（*P*≥0.05）。

**表1:** 42例接受异基因造血干细胞移植伴TP53基因异常骨髓增生异常综合征/急性髓系白血病患者临床资料

指标	TP53缺失组（11例）	TP53单基因突变组（23例）	TP53多打击组（8例）	统计量	*P*值
性别（例，男/女）	6/5	17/6	7/1	2.618	0.270
年龄［岁，*M*（范围）］	38.5（23~53）	41.5（18~62）	25.5（19~46）	0.395	0.676
疾病				0.982	0.612
MDS	6（28.57）	10（47.62）	5（23.81）		
AML	5（23.81）	13（26.00）	3（14.29）		
初诊血细胞计数[*M*（范围）]					
WBC（×10^9^/L）	6.73（1.50~204.00）	3.02（1.32~115.40）	4.48（1.58~99.29）	0.555	0.661
HGB（g/L）	70(48~102)	76（40~122）	85（56~107）	0.540	0.587
PLT（×10^9^/L）	49（7~854）	57.5（14~204）	23.5（11~72）	1.466	0.210
ANC（×10^9^/L）	0.57（0.00~72.3）	1.16（0.28~89.24）	1.28（0.33~87.23）	0.719	0.425
初诊骨髓原始细胞［%，*M*（范围）］					
涂片	13.5（1.0~87.5）	25.5（0.0~97.0）	3.5（2.0~42.5）	1.057	0.345
流式细胞术	26.35（2.70~90.20）	5.00（0.00~88.55）	4.52（0.00~33.30）	2.038	0.089
染色体核型［例（%）］				4.015	0.404
正常核型	4（40.00）	7（30.43）	1（12.50）		
复杂核型	2（20.00）	3（13.04）	4（50.00）		
其他	4（40.00）	11（47.82）	3（37.50）		
−5/5q	1（9.09）	2（8.70）	5（62.50）	9.659	0.008
共突变基因［例（%）］				7.513	0.111
无	3（27.27）	12（52.17）	7（87.50）		
1～2个	4（36.36）	7（30.43）	0（0.00）		
≥3个	4（36.36）	4（17.39）	1（12.50）		
移植前疾病状态［例（%）］				9.113	0.167
NR	5（45.45）	6（26.09）	6（75.00）		
CR	3（27.27）	13（56.52）	2（25.00）		
CRi	3（27.27）	3（13.04）	0（0.00）		
SD	0（0.00）	1（4.35）	0（0.00）		
移植前骨髓原始细胞阳性［例（%）］	5（50.00）	6（35.29）	4（80.00）	3.670	0.160
移植前融合基因阳性［例（%）］	5（55.56）	8（36.36）	5（62.50）	2.029	0.363
供者类型［例（%）］				1.571	0.456
非亲缘	0（0.00）	3（13.04）	1（12.50）		
亲缘	11（100.00）	20（86.96）	7（87.50）		
ABO血型［例（%）］				0.039	0.981
相合	5（45.45）	11（47.83）	4（50.00）		
主/次不合	6（54.55）	12（52.17）	4（50.00）		
CD34^+^细胞回输量［×10^6^/kg，*M*（范围）］	2.81（0.29~5.31）	2.34（1.80~5.58）	2.36（0.51~4.80）	0.072	0.930
中性粒细胞植入时间［d，*M*（范围）］	13（10~20）	12.5（10~21）	12.5（11~16）	0.313	0.969
血小板植入时间［d，*M*（范围）］	19（12~30）	17.5（8~44）	21（14~38）	0.185	0.832
急性GVHD［例（%）］	6（54.55）	14（60.87）	5（62.50）	0.075	0.923
慢性GVHD［例（%）］	4（36.36）	5（21.74）	3（37.50）	1.166	0.558
移植后并发症［例（%）］	1（8.33）	7（30.43）	2（28.57）	1.062	0.352
移植后骨髓原始细胞阳性［例（%）］	2（18.18）	4（17.39）	6（75.00）	10.441	0.005
复发［例（%）］				0.894	0.639
是	2（16.67）	6（26.09）	3（42.86）		
否	10（83.33）	16（69.57）	4（57.14）		
植入失败［例（%）］	0（0.00）	1（4.35）	0（0.00）		
死亡［例（%）］	3（20.00）	9（60.00）	3（20.00）	4.257	0.119
死亡原因［例（%）］				3.533	0.473
复发	2（16.67）	5（21.74）	3（42.86）		
GVHD	0（0.00）	4（17.39）	1（12.50）		

**注** MDS：骨髓增生异常综合征；AML：急性髓系白血病；−5/5q：5号染色体或5号染色体长臂缺失；CR：完全缓解；CRi：严格意义的完全缓解；NR：未缓解；SD：疾病稳定；GVHD：移植物抗宿主病

对患者的移植前疾病状态进行评估，三组移植前未缓解患者分别为5例（45.5％）、6例（26.1％）、6例（75％）。42例患者中38例（90.5％）接受了亲缘供者移植，亲缘供者移植中单倍体与全相合移植各占50.0％（19例）。患者回输CD34^+^细胞数中位值为2.81（0.29～5.31）×10^6^/kg，三组中性粒细胞中位植入时间分别为12、13、12.5 d，血小板中位植入时间分别为21、20、23 d，差异无统计学意义（*P*≥0.05）。42例患者移植后有11例（26.2％）复发，移植后15例（35.7％）患者死亡，复发为主要死亡原因（66.7％，10/15）。

二、总体及各亚组患者allo-HSCT后生存与复发情况

移植后3年OS率为66.3％（95％*CI* 53.4％～82.4％），EFS率为61.0％（95％*CI* 47.7％～78.0％）（图1）。TP53缺失组、TP53单基因突变组、TP53多打击组移植后3年OS率分别为82.5％（95％*CI* 63.1％～100.0％）、60.6％（95％*CI* 43.5％～84.4％）、57.1％（95％*CI* 30.1％～100.0％）（*χ*^2^＝3.100，*P*＝0.200）（图2A），EFS率分别为80.8％（95％*CI* 60.0％～100.0％）、55.0％（95％*CI* 37.8％～80.3％）、50.0％（95％*CI* 25.0％～100.0％）（*χ*^2^＝0.900，*P*＝0.600）（图2B）。

**图1:**
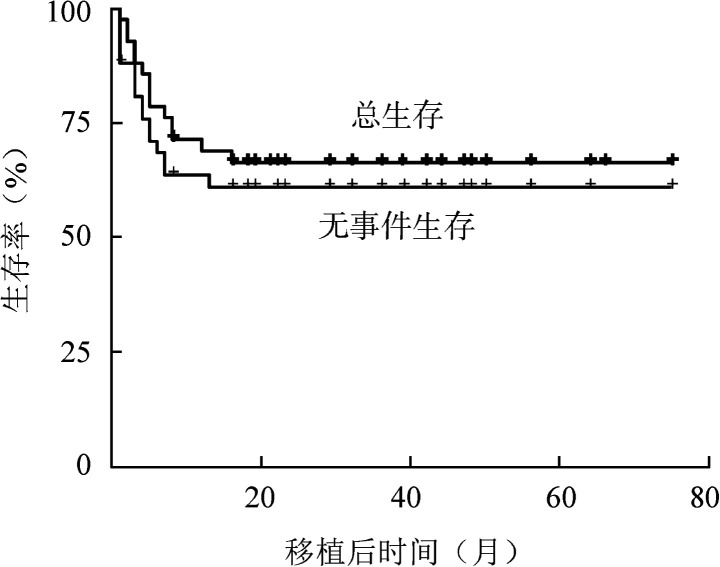
伴TP53基因异常骨髓增生异常综合征/急性髓系白血病患者异基因造血干细胞移植后总生存曲线和无事件生存曲线

**图2:**
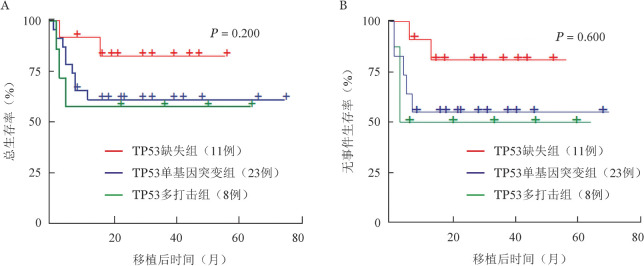
TP53基因异常骨髓增生异常综合征/急性髓系白血病患者异基因造血干细胞移植后总生存曲线（A）和无事件生存曲线（B）

三、预后危险因素分析

随访截至2022年3月31日，将性别、移植年龄、疾病类型、移植前病程、白细胞计数、血小板计数、中性粒细胞计数、骨髓原始细胞比例、TP53基因状态、复杂核型、−5/5q、共突变基因、治疗、移植前疾病状态、移植前骨髓原始细胞阳性、移植前WT1融合基因阳性、HLA配型、急性GVHD、慢性GVHD、移植后骨髓原始细胞阳性、移植后WT1融合基因阳性等因素纳入单因素分析，将单因素分析中*P*<0.1的因素纳入多因素分析，以双侧*P*<0.05为差异有统计学意义。结果显示：年龄、染色体核型、共突变、移植前骨髓原始细胞阳性、移植后骨髓原始细胞阳性为OS、EFS的共同预后不良因素。除此之外，移植前疾病状态也是EFS的预后不良因素。其中移植前原始细胞阳性是OS的独立危险因素（*P*＝0.037，*HR*＝33.40，95％*CI* 1.24～901.17），移植后原始细胞阳性是EFS的独立危险因素（*P*＝0.039，*HR*＝97.79，95％*CI* 1.26～7732.15）。详见表2、3。

**表2:** TP53基因异常骨髓增生异常综合征/急性髓系白血病患者异基因造血干细胞移植后总生存危险因素分析

影响因素	单因素分析		多因素分析	
	*P*值	*HR*（95%*CI*）	*P*值	*HR*（95%*CI*）
移植时年龄	0.059	1.05（1.00~1.11）	0.451	0.96（0.87~1.06）
复杂染色体核型	0.010	2.97（1.30~6.82）	0.144	3.77（0.64~22.33）
共突变	0.033	0.36（0.14~0.92）	0.656	1.94（0.11~35.33）
移植前骨髓原始细胞阳性	0.021	6.20（1.31~29.30）	0.037	33.40（1.24~901.17）
移植后骨髓原始细胞阳性	0.005	4.53（1.57~13.10）	0.051	29.06（0.99~856.98）

**表3:** TP53基因异常骨髓增生异常综合征/急性髓系白血病患者异基因造血干细胞移植后无事件生存危险因素分析

影响因素	单因素分析		多因素分析	
	*P*值	*HR*（95%*CI*）	*P*值	*HR*（95%*CI*）
移植时年龄	0.028	1.06（1.01~1.11）	0.246	1.08（0.95~1.23）
复杂核型	0.002	3.75（1.61~8.71）	0.052	4.57（0.99~21.12）
共突变	0.072	0.50（0.24~1.06）	0.137	19.37（0.39~959.19）
移植前疾病状态	0.067	0.48（0.22~1.05）	0.056	0.05（0.00~1.08）
移植前骨髓原始细胞阳性	0.024	4.55（1.23~16.90）	0.147	9.20（0.46~183.88）
移植后骨髓原始细胞阳性	0.001	5.71（2.10~15.60）	0.039	97.79（1.26~7732.15）

四、移植前原始细胞阳性对移植后生存的影响

移植前原始细胞阴性组（27例）、阳性组（15例）移植后3年OS率分别为87.4％（95％*CI* 72.4％～100.0％）、46.7％（95％*CI* 27.2％～80.2％）（*χ*^2^＝6.686，*P*＝0.009，图3A），3年EFS率分别为80.5％（95％*CI* 62.8％～100.0％）、40.0％（95％*CI* 21.5％～74.3％）（*χ*^2^＝6.221，*P*＝0.014，图3B）。TP53单基因突变组（23例）中，移植前骨髓原始细胞阴性（6例）、阳性（17例）患者移植后3年OS率分别为90.9％、33.3％（*χ*^2^＝3.584，*P*＝0.058）。

**图3:**
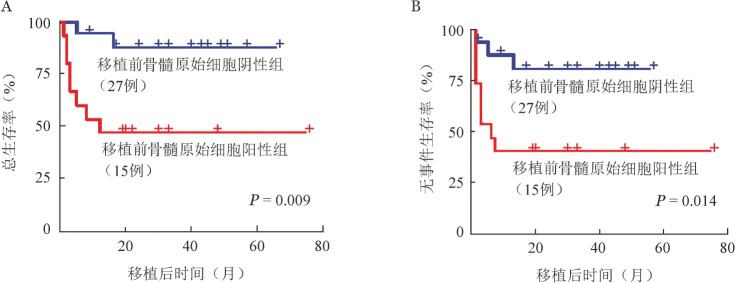
移植前骨髓原始细胞阴性、阳性伴TP53基因异常骨髓增生异常综合征/急性髓系白血病患者移植后总生存曲线（A）与无事件生存曲线（B）

五、复杂核型对移植后生存的影响

正常核型组（12例）、复杂核型组（9例）移植后3年OS率分别为91.7％（95％*CI* 77.3％～100.0％）、38.1％（95％*CI* 19.6％～74.2％）（*χ*^2^＝8.261，*P*＝0.004），3年EFS率分别为90.9％（95％*CI* 75.4％～100.0％）、66.7％（95％*CI* 42.0％～100.0％）（*χ*^2^＝4.345，*P*＝0.032）。

## 讨论

对相关研究进行回顾发现，既往报道的伴有TP53异常的MDS/AML患者接受allo-HSCT后的OS各不相同。Britt等[13]在一项回顾性研究中发现伴17号染色体改变的MDS/AML患者移植后生存较差，其中同时伴有TP53基因突变和−17患者的移植后3年OS率不足20％，考虑这可能与患者构成比有关。张莹等[14]总结了37例伴TP53突变的MDS/AML患者接受allo-HSCT后疗效分析，发现患者2年OS率不足40％，这可能与19例患者接受了降低强度预处理（RIC）方案相关。本组TP53基因异常（包括单突变、缺失、多打击）MDS/AML患者allo-HSCT后3年OS率为66.3％，EFS率为61.0％，显示allo-HSCT可以克服TP53基因异常对预后带来的不良影响。与以往研究相比，本组病例allo-HSCT后生存较好，这与可能与不同研究之间预处理方案、患者疾病分布差异等因素有关。

本研究比较了不同TP53基因异常MDS/AML患者allo-HSCT后的生存情况，尽管差异无统计学意义（*P*≥0.05），但发现TP53缺失组患者较TP53单基因突变组以及TP53多打击组有预后更佳的趋势，3年OS率达82.5％。Boettcher等[15]通过建立TP53细胞系模型，对比TP53野生型、TP53纯和缺失型及TP53突变型细胞系的表型及功能，发现TP53突变显著抑制了正常P53蛋白转录下游基因P21的能力，提示TP53突变发挥显性负效应是血液系统恶性肿瘤中TP53突变是导致不良预后的重要原因。

本研究结果显示移植前骨髓原始细胞阳性是影响OS的独立危险因素，移植前阴性的患者较阳性患者具有更好的移植后3年OS率（87.4％对46.7％），提示移植前缓解状态较好的患者预后更佳。目前，移植前残留阳性是AML患者移植预后的不良预后因素已成为共识。但对于MDS患者，目前我国以及NCCN指南均提示移植前降细胞治疗并不改善患者预后。本研究结果提示伴有TP53异常的MDS患者移植前骨髓原始细胞阴性是预后良好的因素，患者可能受益于较低肿瘤负荷的良好缓解状态。这也提示对MDS患者移植时机的把握可能需要通过对患者危险度及疾病特征进行分层，从而进一步精准制定治疗方案。但受限于样本量，研究结果需进一步进行验证。

同时，尽管我们的研究中未发现移植前的疾病状态是影响OS和EFS的危险因素，但有研究认为移植前疾病未缓解是影响移植后OS的危险因素[16]，并指出一线治疗后患者疾病是否缓解与移植后疗效有较强的相关[17]。Sallman等[18]比较了接受去甲基化治疗、强化疗、移植的疗效，提出免疫治疗策略可能使该分子学特异性亚群受益，但仅观察到一小部分MDS/SAML患者对免疫检查点治疗的应答，缺乏更进一步的亚组分析。一项比较接受HMA治疗后患者TP53差异甲基化区（DMR）水平的研究结果显示，在接受HMA治疗的患者中，高DMR的患者仍多于低DMR的患者[19]，提示临床应用去甲基化药物治疗以及如何检测疾病甲基化水平仍值得进一步探讨。

本研究中TP53基因异常伴复杂染色体核型患者较伴正常染色体核型患者显示出了较差的预后，尤其是在同时存在TP53基因多打击改变患者组，此部分患者可能是未来临床研究需要攻克的难题。尽管样本量较少，但本研究结果与国内张莹等[20]报道相似。Haase等[21]发表的一项对伴有复杂染色体核型MDS（CK-MDS）进行预后分层的研究表明，大约10％的MDS患者伴有复杂核型，TP53基因突变存在于55％的CK-MDS患者中，认为CK-MDS患者的不良预后是由TP53基因突变驱动发生的。以往研究已证明残留的野生型TP53基因对于维持基因组的稳定性至关重要，伴有TP53基因突变肿瘤患者基因组的扩增和深度缺失率比野生型TP53高出约2.5倍[15],[17]。TP53单等位基因突变与双等位基因靶向改变指向不同的进化轨迹或克隆优势。前者更易伴有多个其他基因突变；后者更具有克隆间竞争和获得TP53克隆的克隆优势，指向复杂核型，更难以保持基因组的稳定性。

本研究结果显示allo-HSCT可改善伴TP53基因异常MDS/AML患者的生存，但伴复杂核型TP53基因异常MDS/AML患者预后不佳。本研究为单中心、回顾性分析且病例较少，以上结论尚需多中心、大样本研究加以验证。
